# Sphingomyelinase D/Ceramide 1-Phosphate in Cell Survival and Inflammation

**DOI:** 10.3390/toxins7051457

**Published:** 2015-04-29

**Authors:** Io-Guané Rivera, Marta Ordoñez, Natalia Presa, Ana Gomez-Larrauri, Jorge Simón, Miguel Trueba, Antonio Gomez-Muñoz

**Affiliations:** Department of Biochemistry and Molecular Biology, Faculty of Science and Technology, University of the Basque Country (UPV/EHU), 48080 Bilbao, Spain; E-Mails: io_rivera@hotmail.com (I.-G.R.); marta.ordonez87@gmail.com (M.O.); npretor@gmail.com (N.P.); anagola@hotmail.com (A.G.-L.); jorgesimon04@gmail.com (J.S.); miguel.trueba@ehu.es (M.T.)

**Keywords:** cell survival, cell migration, ceramides, ceramide 1-phosphate, loxoscelism, inflammation, sphingolipids, sphingomyelin D

## Abstract

Sphingolipids are major constituents of biological membranes of eukaryotic cells. Many studies have shown that sphingomyelin (SM) is a major phospholipid in cell bilayers and is mainly localized to the plasma membrane of cells, where it serves both as a building block for cell architecture and as a precursor of bioactive sphingolipids. In particular, upregulation of (C-type) sphingomyelinases will produce ceramide, which regulates many physiological functions including apoptosis, senescence, or cell differentiation. Interestingly, the venom of some arthropodes including spiders of the genus *Loxosceles*, or the toxins of some bacteria such as *Corynebacterium tuberculosis*, or *Vibrio damsela* possess high levels of D-type sphingomyelinase (SMase D). This enzyme catalyzes the hydrolysis of SM to yield ceramide 1-phosphate (C1P), which promotes cell growth and survival and is a potent pro-inflammatory agent in different cell types. In particular, C1P stimulates cytosolic phospholipase A2 leading to arachidonic acid release and the subsequent formation of eicosanoids, actions that are all associated to the promotion of inflammation. In addition, C1P potently stimulates macrophage migration, which has also been associated to inflammatory responses. Interestingly, this action required the interaction of C1P with a specific plasma membrane receptor, whereas accumulation of intracellular C1P failed to stimulate chemotaxis. The C1P receptor is coupled to Gi proteins and activates of the PI3K/Akt and MEK/ERK1-2 pathways upon ligation with C1P. The proposed review will address novel aspects on the control of inflammatory responses by C1P and will highlight the molecular mechanisms whereby C1P exerts these actions.

## 1. Introduction

**Figure 1:**
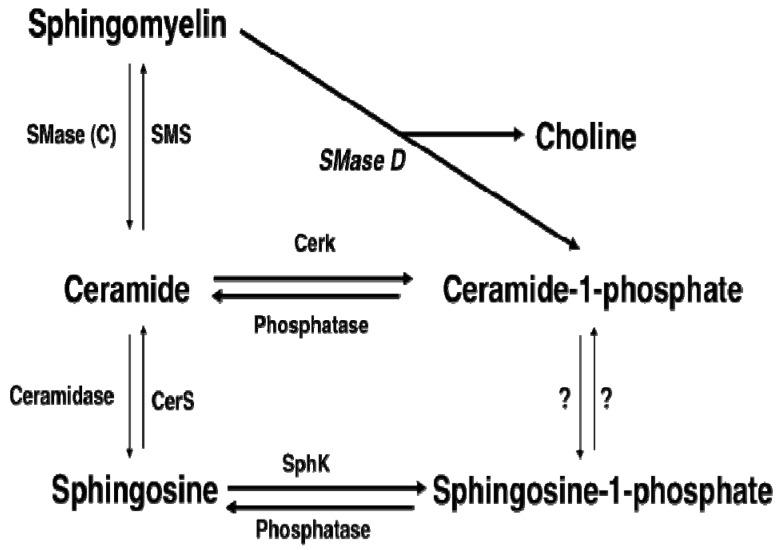
Ceramide 1-phosphate production by sphingomyelinase activities. C1P can be generated directly by the action of SMase D acting on SM. This pathway may not exist in mammalian cells. C1P can be indirectly synthesized by the action of C-type sphingomyelinase activities, which generate ceramide, in combination with ceramide kinase (CerK) activity. This is a major pathway for C1P formation in mammalian cells. C1P can also be synthesized from ceramide produced by *de novo* synthesis, which is another major pathway for synthesis of C1P in mammalian cells. SMase, sphingomyelinase; SphK, sphingosine kinase; CerS, ceramide synthase.

Sphingomyelinase D (SMase D) was identified as the active component of the venom of spiders of the *Sicariid* family. This family includes spiders of the genus *Loxosceles* (violin or fiddleback spiders) and *Sicarius* (six-eyed sand spiders). The most widespread and important *Loxosceles* species in different areas of the World are *L*. *laeta*, *L. reclusa*, *L. rufescens*, *L. arizonica*, *L. intermedia* and *L. gaucho*. The latter were shown to be the cause of envenomation in humans. The brown spider, *L. reclusa*, is native to the United States, where it can be found in south and central states. *L. laeta* probably originated from west South America, and *L. rufescens* has been recorded in Spain. In fact, *L. rufescens* is the most poisonous spider in the Iberia peninsula [1]. It is now well established that SMase D is the cause for dermonecrotic skin lesions in humans bitten by *Loxosceles* arachnids, and is the main component of the venom responsible for the local and systemic effects observed in loxoscelism. In particular, *Loxosceles* envenomation resulted in the production of pro-inflammatory cytokines such as IL-8, and GRO-alpha, or chemokines including monocyte chemoattractant protein-1 (MCP-1). In human umbilical vein endothelial cells, *Loxosceles* venom-induced chemokine or cytokine expression is mediated by the nuclear factor kappa B (NF-kB) [2]. SMase D has not been detected in mammalian cells. However, although this enzyme is unknown elsewhere in the animal kingdom, its presence in nature is not restricted to arachnids, as it is also a major component of the toxins of some bacteria, including *Corynebacterium pseudotuberculosis*, *C. ulcerans*, *Archanobacterium haemoliticum*, and *Vibrio damsela* [3]. It was hypothesized that the primary functional role of SMase D is to help with immobilizing or pre-digesting arthropodes, rather than to cause lesions in mammals [1]. SMase D catalyzes the cleavage of sphingomyelin, a phospholipid that is present in the plasma membrane of cells, where it serves both structural and functional roles. The two major products of SMase D activity are ceramide 1-phosphate (C1P) and choline (Figure 1). Whereas the latter is a biologically inert metabolite, C1P is biologically active. Specifically, C1P was shown to potently stimulate cell proliferation [4,5,6,7,8,9,10], block cell death through inhibition of apoptosis [11,12,13], induce cell migration [14,15], and promote inflammation [16,17,18,19]. Of interest, SMase D is also very active in hydrolyzing lysophosphatidylcholine (LPC) to release lysophosphatidic acid (LPA), but not phosphatidylcholine (PC) [20]. This was later confirmed by Lee and Lynch who found that the recombinant enzyme from *Loxosceles laeta* cleaves LPC to LPA and choline [21]. Therefore, C1P may not be the only active component of these spider venoms or bacterial toxins.

LPA is a pleiotropic growth factor-like lipid mediator that has been shown to induce various pathophysiological effects including platelet aggregation, cytokine and chemokine secretion, endothelial hyperpermeability, chemotaxis, inflammation, angiogenesis, and tumor progression [22,23,24]. LPA mediates many of its effects through activation of three Gi protein-coupled receptors of the endothelial differentiation gene family, now known as LPA1, LPA2 and LPA3. More recently, the nuclear transcription factor peroxisome proliferator-activator receptor-gamma (PPARγ) was identified as an intracellular receptor for LPA [25]. The generation of LPA by the action of SMase D acting on LPC has been suggested not to be physiological, as the Km value of the enzyme is relatively high (around 8 mM) [20]. However, van Meeteren and co-workers [22] found that spider and bacterial SMase D possess intrinsic lyso-PLD activity toward albumin-bound LPC, and that expression of functional LPA receptors is necessary and sufficient for at least some of the biological responses to SMase D. The latter authors conclude that degradation of circulating LPC to LPA may significantly contribute to the pathogenecity of SMase D [22].

## 2. Biological Activities of C1P

### 2.1. C1P Promotes Cell Proliferation and Survival

C1P was first reported to be mitogenic for different cell types, including fibroblasts [5,6], macrophages [4,7,8,10], and myoblasts [9], and was later found to promote cell survival [11,12,13]. However, C1P can be deleterious for cells at high concentrations [10,13]. The major mechanisms by which C1P elicited its mitogenic effects included stimulation of the MEK/ERK and PI3K/Akt pathways, as well as activation of p38, mammalian target of rapamycin (mTOR), protein kinase C-alpha (PKC-α), ROCK/RhoA, retinoblastoma, and small increases in reactive oxygen species (ROS) [7,8,10,26]. In this connection, we recently reported that ceramide kinase (CerK), the enzyme responsible for C1P generation in mammalian cells, is essential for proliferation of human neuroblastoma cells, as downregulation of this enzyme with specific gen silencing siRNA or with pharmacological inhibitors, dramatically reduced neuroblastoma cell proliferation [27]. CerK was also implicated in the stimulation of cell proliferation and inhibition of apoptosis in human A459 lung adenocarcinoma cells [28], thereby confirming the role of C1P in controlling cell growth and death. Moreover, Murakami and co-workers observed that CerK mRNA levels were reduced during all-trans retinoic acid (ATRA)-induced differentiation of human neuroblastoma cells [29], an action that involved inhibition of the transcriptional activity of the 5'-promoter of CerK. The promotion of cell survival by C1P involved inhibition of acid sphingomyelinase (ASMase) [11] or serine palmitoyltransferase (SPT) [13] with subsequent decrease in ceramide levels, inhibition of caspases 9 and 3, as well as activation of inducible nitric oxide synthase (iNOS) expression and the subsequent elevation of nitric oxide (NO) levels [12]. Many of the above studies were performed using exogenous C1P to stimulate the cells. Although cell membranes are not very permeable to C1P, we observed that it can be incorporated by cells [6,9]. This should not be surprising when taken into consideration the existence of a specific C1P transporter that is present in the cytosol and different cellular membrane compartments including the plasma membrane of cells [30]. Also, like for other phospholipids, C1P can cross the plasma membrane by flip-flop mechanisms. The ubiquitously expressed C1P transfer protein was named CPTP, and was shown to specifically transfer C1P between membranes. Interestingly, it was postulated that CPTP could transfer C1P to an intracellular site where it exerts an inhibitory effect on SM degradation [30], a situation that is in agreement with the inhibition of ASMase by C1P [11]. In fact, downregulation of CPTP caused a decrease in C1P trafficking and prevented ASMase inhibition. To elucidate whether the effects of C1P on cell proliferation and survival were caused by intracellular C1P, photosensitive caged C1P analogues were used. Specifically, 7-(*N*,*N*-diethylamino) coumarin (DECM-C1P), and 4-bromo-5-hydroxy-2-nitrobenzhydryl moiety (BHNB-C1P) are cell permeable and biologically inactive. These compounds bypass cell receptors and can easily cross the plasma membrane [31]. Once inside the cells C1P can be uncaged through irradiation with visible light that does not harm cellular components (400–500 nm wavelength light) thereby releasing free C1P into the cytosol. This experimental approach confirmed that intracellular C1P was responsible for stimulation of cell proliferation in macrophages, at relatively low concentration [31]. Ongoing experiments in our laboratory also indicate that C1P is responsible for inhibiting cell death, when used at relatively low concentrations (5–30 µM). However, higher concentrations of C1P cause cell death, a finding that is in agreement with the induction of dermonecrotic lesions after *Loxosceles* bites, where local concentrations of SMaseD/C1P in skin areas surrounding the bite may be very high.

### 2.2. Implication of C1P in Inflammatory Responses

The biological effects of the venom of *Loxosceles* spiders are consistent with the pro-inflammatory effects elicited by C1P in mammalian cells. It was first reported that C1P stimulates arachidonic acid (AA) release and the subsequent synthesis of prostaglandins in human A549 lung adenocarcinoma cells [32]. This action involved direct interaction of C1P with its amino terminal CaLB domain (also known as C2 lipid binding domain) of cytosolic phospholipase A2 (cPLA_2_) leading to its activation [33,34]. On the other hand, C1P was shown to inhibit tumor necrosis factor (TNF)-converting enzyme, also known as TACE or ADAM 17, which is the major matrix metalloproteinase responsible for cleavage of pro-TNF to release the active inflammatory form [35]. It was speculated that C1P might be metabolized to sphingosine 1-phosphate (S1P) to elicit its pro-inflammatory actions. However, C1P is slowly metabolized in cells, and no significant amounts of S1P could be detected after several hours of challenging cells with exogenous C1P [5,9,19,36,37]. Nonetheless, C1P was shown to act in coordination with S1P to maximize the production of prostaglandins. In particular, S1P stimulates cyclooxigenase-2 (COX-2) activity, which then uses the AA produced by cPLA2 as substrate to synthesize prostaglandins [38]. In addition, C1P has been shown to control inflammatory responses through stimulation of phagocytosis in neutrophils [39,40], and activation of degranulation in mast cells [41]. In addition, CerK knockout mice showed less inflammation when fed a high fat diet thereby implicating this enzyme in obesity-associated inflammation [42].

### 2.3. Ceramide 1-Phosphate Promotes Cell Migration

Immune cells including lymphocytes and macrophages, migrate from the blood stream to the sites of lesions or infection to protect the cells and to help repair the damaged tissue, thereby promoting inflammation. These processes involve upregulation of cytokines, chemokines, extracellular matrix proteinases, or integrins. Although it is clear that cytokines such as macrophage colony stimulating factor (M-CSF), chemokines such as monocyte chemotactic protein-1 (MCP-1), or integrins play significant roles in regulating adhesion and migration of macrophages and neutrophils, the mechanisms or signaling pathways responsible for coordinating these processes have not been well characterized. In this connection, we have demonstrated that C1P potently induces macrophage migration [14]. Noteworthy, this action was only observed when C1P was administered exogenously to the cells, and not by increasing the intracellular levels of C1P. This observation suggested the existence of a C1P receptor responsible for this novel action of C1P. Radioligand binding assays using [^3^H]C1P or [^33^P]C1P and isolated macrophage cell membranes resulted in identification of a specific receptor through which C1P stimulated cell migration. This receptor specifically bound C1P, but not other sphingolipids, and was sensitive to inhibition by pertussis toxin, a G_i_ protein inhibitor. The receptor had a low affinity for its ligand (apparent *K*_d_ for C1P was about 7.7 µM) and the Bmax value was about 1269 pmol/mg protein. Ligation of the receptor by C1P triggered rapid phosphorylation of ERK1-2, c-JNK, as well as stimulation of the PI3K/PKB (Akt) pathway. Inhibition of any of these pathways using specific siRNA technology or selective chemical inhibitors abolished C1P-stimulated macrophage migration. In addition, C1P stimulated the activity of NF-κB, and inhibition of this transcription factor also led to blockade of macrophage migration. Hence, these findings suggest that activation of MEK/ERK1-2, PI3-K/PKB (Akt) and NF-κB are major mechanisms by which C1P stimulates cell migration. The pro-chemotactic effects of C1P have been recently confirmed in other cell types. In particular, we found that C1P stimulated migration of human acute monocytic leukemia THP-1 cells, or 3T3 pre-adipocytes [15], and Kim and co-workers [43] found that C1P is a potent chemoattractant for homing of hematopoietic stem/progenitor cells to the bone marrow. In addition, the same group demonstrated that C1P is a major regulator of multipotent stromal cell and endothelial progenitor cell migration [44], and that S1P and C1P stimulate migration of bone marrow derived stem cells in patients suffering from acute myocardial infarction [45]. The latter observations place C1P as central regulator of tissue regeneration, and implicate C1P in the promotion of tumor dissemination [44]. Of interest, our recent work indicates that phosphatidic acid (PA), which is a lipid precursor for phospholipid and triacylglycerol biosynthesis, and is also a signaling metabolite, can also bind to the C1P receptor to counteract C1P-stimulated cell migration [46]. In addition, C1P-stimulated macrophage migration was inhibited by treatment of the cells with exogenous phospholipase D, the enzyme responsible for generation of PA at the plasma membrane, suggesting that PA may be a physiological regulator of macrophage migration.

Although initial studies revealed that C1P has pro-inflammatory properties, recent investigation suggests that it can also exert anti-inflammatory actions under certain circumstances, and that C1P can preferentially induce pro- or anti-inflammatory processes depending on cell type. For instance, in human peripheral blood mononuclear cells, C1P was found to suppress lipopolysaccharide-mediated production of pro-inflammatory cytokines, including TNF, interleukin (IL)-6, IL-8 and IL-1β, and inhibited TNF-α converting enzyme (TACE), which is the enzyme responsible for production of TNF-α [35]. Noteworthy, C1P has been shown to be anti-inflammatory in lung tissue. Specifically, intrapulmonary administration of C1P reduced cigarette smoke-induced acute lung inflammation and development of emphysema [47]. Also, accumulation of ceramides derived from the action of ASMase was shown to play an important role in pulmonary infections as ceramides facilitate internalization of bacteria into lung epithelial cells [48]. Of interest, it was demonstrated that PAF induces pulmonary edema by a mechanism involving A-SMase activation and the subsequent production of ceramides [49]. In this context, the inhibition of A-SMase by C1P that we previously observed [11] could be important to reduce inflammation in the lung. Although this would be a beneficial effect of C1P in lung pathophysiology, a putative anti-inflammatory effect of SMase D in the lung remains to be established.

## 3. Concluding Remarks

SMase D, contained in the venom of some types of spiders or the toxins of some bacteria, is a promiscuous enzyme that uses various lipid substrates to elicit its toxic effects in eukaryotic cells. A major product of SMase D activity is C1P, which can induce cell proliferation, and cell migration and is involved in inflammation. Participation of C1P in inflammatory responses is controversial as it can induce pro-inflammatory actions in different cell types, including macrophages, but can also counteract inflammation in lung tissue. SMase D can efficiently hydrolyze phospholipids other than SM including LPC or LPI to produce LPA, which like C1P, can also promote cell migration and inflammation. Both SMase D products, C1P and LPA, have been shown to elicit some of its effects through interaction with specific Gi protein-coupled receptors thereby causing their toxic effects through the action of different mechanisms working on the surface of eukaryotic cell membranes. However, in contrast to LPA or S1P, a receptor for C1P remains to be characterized. This scenario becomes more complicated when taking into account that the effects of C1P depend on the composition of the cell membrane, and so SMase D may elicit different effects in different cell types. Further investigation into the biological effects of C1P will help understand the molecular mechanisms involved in loxoscelism and infections caused by SMase D-containing bacterial toxins.
